# Modeling Illicit Drug Use Dynamics and Its Optimal Control Analysis

**DOI:** 10.1155/2015/383154

**Published:** 2015-12-27

**Authors:** Steady Mushayabasa, Gift Tapedzesa

**Affiliations:** University of Zimbabwe, Department of Mathematics, P.O. Box MP 167, Harare, Zimbabwe

## Abstract

The global burden of death and disability attributable to illicit drug use, remains a significant threat to public health for both developed and developing nations. This paper presents a new mathematical modeling framework to investigate the effects of illicit drug use in the community. In our model the transmission process is captured as a social “contact” process between the susceptible individuals and illicit drug users. We conduct both epidemic and endemic analysis, with a focus on the threshold dynamics characterized by the basic reproduction number. Using our model, we present illustrative numerical results with a case study in Cape Town, Gauteng, Mpumalanga and Durban communities of South Africa. In addition, the basic model is extended to incorporate time dependent intervention strategies.

## 1. Introduction

Illicit drug use continues to exert significant toll, with valuable human lives and productive years of many people being lost. An estimated 183,000 drug-related deaths were reported in 2012 [1]. Globally, it is estimated that, in 2012, between 162 million and 324 million people aged between 15 and 64 had used an illicit drug [1]. Illicit drug use is defined as the nonmedical use of a variety of drugs that are prohibited by international law [2]. These drugs include amphetamine-type stimulants, cannabis, cocaine, heroin, and other opioids, and MDMA (ecstasy) [3]. The risk of premature mortality and morbidity from illicit drug use is dependent on dose, frequency, and route of administration [2]. Further, the mortality risks of illicit drug consumption increase with increasing frequency and quantity of consumption [4]. The first global burden of death and disability attributable to illicit drug use was first estimated by Donoghoe [5], as part of the global burden of disease project [6]. Donoghoe estimated that illicit drug use was responsible for 10,000 deaths globally in 1990 with about 62% of them in developing countries [6]. Besides causing deaths, illicit drug use has been associated with 50% of mental illness cases [7].  Table 1 illustrates the cumulative prevalence of illicit drug use in Cape Town, South Africa, from 1996 (June) to 2008 (June); the data was adopted from SACENDU [8].

Additional data on recorded cases of illicit drug use in some communities of South Africa, namely, Durban (Table 4), Gauteng (Table 5), and Mpumalanga (Table 6), is presented in Appendix A.

Despite many clinical and theoretical studies [9–20] and tremendous educational campaigns [1, 7, 21, 22], illicit drug use remains a significant threat to public health all over the world. The use of mathematical models to explore the dynamics of drug use has been an interesting topic for a couple of researchers [11–13]. In 2007, White and Comiskey [13] proposed a mathematical model to evaluate the role of treatment and relapse in the dynamics of heroin. Their work revealed among others that relapse of individuals who would have quit heroin use has a significant impact on the generation of new or secondary heroin users. Most recently, Nyabadza et al. [11] constructed a mathematical model to examine the dynamics of crystal meth “Tik” abuse in the presence of drug-supply chains. Using the data from South Africa, their work suggests among others that programs aimed at encouraging light drug users to quit drug use can be more effective to control “Tik” abuse in South Africa.

Our objective in this paper is to formulate a mathematical model for illicit drug use that includes relevant biological and social aspects, accounts for case detection of drug users, and allows optimal control methods to be used. We stress that our model is not for a specific substance abuse. To begin with, we integrate the aforementioned essential components into one SIR-type (Susceptible-Infectious (individuals who are drug users)-Recovered (individuals in rehabilitation)) model to accommodate the diverse dynamics of illicit drug use determined by population specific parameters such as the rates of light drug user to heavy drug user, light drug user to mental illness, and heavy drug user to mental illness. Our study differs from those in the literature in the fact that it accounts for individuals who become mentally ill due to illicit drug use. It is worth noting that the stigma attached to substance abuse and mental disorders often hinders early detection, diagnosis, and proper treatment [7]. We are aware that mental illness can lead to drug abuse. However, in this paper, we consider that individuals become mentally ill due to illicit drug use.

After comprehensive mathematical analysis, we extend the basic model to incorporate two control functions: the goal of the first control function *u*(*t*) is to reduce the intensity of “social influence,” that is, to weaken the intensity of social interaction between the susceptible population and illicit drug users, while the second *v*(*t*) attempts to increase the rate of detection and rehabilitation of illicit drug users. Optimal control theory has found wide-ranging applications in solving biological problems [23] and network structures [24]. Numerical results followed by a brief discussion round up the paper.

## 2. Mathematical Model

### 2.1. Model Framework

A population of size *N*(*t*) is partitioned into subclasses of susceptible individuals *S*(*t*) (individuals who are not yet illicit drug users but interact with drug users), light or occasional drug users *I*(*t*), heavy drug users *I*_*a*_(*t*), mentally ill population *M*(*t*) (individuals who suffer mental illness due to drug use), and detected illicit drug users *R*(*t*). Thus, *N*(*t*) = *S*(*t*) + *I*(*t*) + *I*_*a*_(*t*) + *M*(*t*) + *R*(*t*). We assume a constant size population with recruitment and non-illicit-related death rate at time *t* given by *μ*. Susceptible individuals acquire illicit drug use habits at rate

(1) $$\begin{array}{c} \lambda =\beta \left(\;I+\kappa I_{a}\;\right). \end{array}$$

The parameter *β* measures the strength of interactions between the susceptible individuals and illicit drug users, that is, the “influence” of *I* and *I*_*a*_ on *S*; *κ* ≥ 1 is a modification factor which accounts for the increased likelihood of heavy illicit drug users *I*_*a*_ to influence more new drug users compared to light drug users *I*. The model takes the form

(2) S˙=μ−λS−μS,I˙=λS−α+γ+σ+μ+ψI,Ia˙=αI−ρ+ϕ+μ+dIa,M˙=σI+ϕIa−ϵ+μ+δM,R˙=γI+ρIa+ϵM−μ+ωR,

where the upper dot represents the derivative of the component with respect to time. The parameter *α* is the rate at which light drug users become heavy drug users; *γ*, *ϵ*, and *ρ* denote the rates of detection and rehabilitation of individuals in classes *I*, *M*, and *I*_*a*_, respectively; *σ* and *ϕ* (*σ* ≤ *ϕ*) denote the rates at which light and heavy illicit drug users develop mental illness; *ψ* and *d* model the permanent exit rates of light and heavy users, respectively, due to either cessation or drug use-related death. Individuals in rehabilitation recover at rate *ω* and are assumed to permanently exit the model. Mentally ill individuals permanently exit the model at rate *δ* due to drug use-related death. Further, we assume that the mentally ill population does not influence the susceptible individuals to become illicit drug users. We study system (2) in the closed set:

(3) $$\begin{array}{c} \Omega =\left\{\;\left(\;S,I,I_{a},M,R\;\right)\in R_{+}^{5}:0\leq N\leq 1\;\right\}, \end{array}$$

where *Ω* is positively invariant with respect to (2). In the absence of drug abuse in the community system (2) admits a drug-free equilibrium point given by

(4) $$\begin{array}{c} E^{0}=\left(\;S^{0},I^{0},I_{a}^{0},M^{0},R^{0}\;\right)=\left(\;1,0,0,0,0\;\right). \end{array}$$

The basic reproductive number provides an invasion criterion for the initial spread of the drug misuse in a susceptible population. For this case, the reproductive number is given by (see computations in Appendix B)

(5) $$\begin{array}{c} R_{d}=\frac{\beta \left(\alpha \kappa +\mu +\rho +\phi +d\right)}{\left(\alpha +\gamma +\mu +\sigma +\psi \right)\left(\mu +\rho +\phi +d\right)}. \end{array}$$

The threshold quantity *ℛ*_*d*_ gives the average number of new drug abusers generated by a typical light or heavy drug user during his or her lifetime as an illicit drug user.

Theorem 1 follows from computations in Appendix C.

**Theorem 1 .**

The drug-free equilibrium point *ℰ* ^0^ is globally asymptotically stable if *ℛ*_*d*_ ≤ 1. If *ℛ*_*d*_ > 1, then a unique drug persistence equilibrium point *ℰ* ^*∗*^ of system (2) exists and is globally asymptotically stable.

### 2.2. Numerical Results

In this section, we estimate the model parameters used in our numerical simulations. In order to carry out the estimation, we made use of the data from SACENDU (see Appendix A). The data has also been used to estimate the best fit for our model.

**Figure 1:**
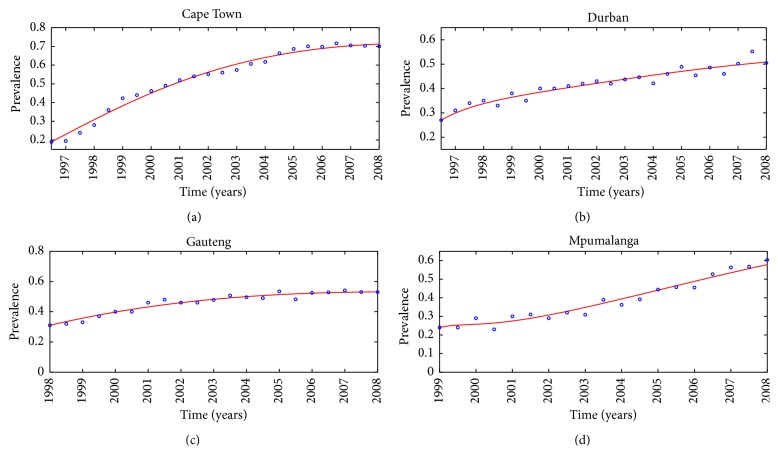
Model system (2) fitted to data for individuals seeking treatment due to illicit drug use. The blue circles indicate the actual data and the solid line indicates the model fit to the data.

Figure 1 shows “best” fit to the real data, illustrated in Appendix A. Here, the fitting process involved the use of the least squares-curve fitting method. A Matlab code has been used together with unknown parameters assigned lower and upper bound from which a set of parameter values that produce the best fit have been obtained. With the exception of Gauteng, simulation results in Figure 1 suggest that the prevalence of illicit drug use increases rapidly over the defined time intervals, while for Gauteng, the increase is gradual. Using the lower bounds of parameter values in Table 2, direct calculation shows that *ℛ*_*d*_ = 6.22, which shows that effective methods should be designed to reduce illicit drug use in these communities. Further, from these simulations, one can suggest that system (C.3) can be an essential tool in predicting future illicit drug use in the communities.

#### 2.2.1. Sensitivity Analysis of the Reproductive Number

Sensitivity analysis of model parameters is very important to design and control strategies as well as a direction to future research. Local sensitivity indices allow us to measure the relative change in a state variable when a parameter changes. In computing the sensitivity analysis, we adopt the approach described by Arriola [25]. The normalized forward sensitivity index of a variable to a parameter is the ratio of the relative change in the variable to the relative change in the parameter. When the variable is a differentiable function of the parameter, the sensitivity index may be alternatively defined using partial derivatives.

**Definition 2 .**

The normalized forward sensitivity index of a variable, *u*, that depends differentiably on a parameter, *p*, is defined as

(6) $$\begin{array}{c} \Gamma_{p}^{u}≔\frac{\partial u}{\partial p}\times \frac{p}{u}. \end{array}$$

To determine the numerical output for our sensitivity indices, we used the lower bounds of parameters in Table 2.  Table 3 presents the model parameters and their sensitivity indices. Model parameters whose sensitivity index values are near −1 or +1 suggest that a change in their magnitude has a significant impact on either increasing or decreasing the size of the reproductive number *ℛ*_*d*_. The results here clearly show that *β* is the most sensitive parameter to *ℛ*_*d*_. An increase in *β* by 10% would increase *ℛ*_*d*_ by 10%. Thus, effective methods aimed at reducing illicit drug and its adverse effects in the community should be designed with the strong view of weakening strength of interactions between the susceptible individuals and illicit drug users.

### 2.3. Optimal Control Problem and Its Analysis

In this section, we introduce two control functions, *u*(*t*) and *v*(*t*), to system (2). The goal of the first control is to reduce the intensity of “social influence” while the second models the effort on the detection of illicit drug users. Thus, system (2) becomes

(7) S˙=μ−1−uλS−μS,I˙=1−uλS−α+γv+σ+μ+ψI,Ia˙=αI−ϕ+ρv+μ+dIa,M˙=σI+ϕIa−μ+ϵv+δM,R˙=vγI+ρIa+ϵM−μ+ωR.

The objective functional, *J*, is used to formulate the relevant optimization problem: finding the most effective strategy that reduces or eliminates the levels of illicit drug use in the community at minimal cost. This minimization goal will be achieved through the implementation of controls *u*(*t*) and *v*(*t*) over the preselected time interval [0, *T*]. Mathematically, this corresponds to the minimization of the functional *J* over a set of feasible, *u*(*t*) and *v*(*t*), strategies on [0, *T*]. *J* functional is defined as follows:

(8) $$\begin{array}{c} J\left(\;u,v\;\right)=\overset{T}{\underset{0}{\int }}\left[\;I+I_{a}+Au^{2}+B_{2}v^{2}\;\right]dt, \end{array}$$

where *A* and *B* are balancing coefficients transforming the integral into dollars expended over a finite time period of *T* years. The balancing coefficients account for the relative size and importance preassigned by the modelers to the contributing terms in the objective functional.

We consider state system (7) of ordinary differential equations in *ℝ* ^5^ with the set of admissible control functions given by

(9) Γ=ut,vt∈L10,T ∣ 0≤ut,  vt≤1,  ∀t∈0,T.

We consider the optimal control problem of determining *S* ^*∗*^(*t*), *I* ^*∗*^(*t*), *I*_*a*_^*∗*^(*t*), *M* ^*∗*^(*t*), and *R* ^*∗*^(*t*), associated with an admissible control pair (*u* ^*∗*^(*t*), *v* ^*∗*^(*t*)) ∈ Γ on the time interval [0, *T*], satisfying (7), given the initial conditions *S*(0), *I*(0), *I*_*a*_(0), *M*(0), and *R*(0), and minimizing the cost functional (8); that is,

(10) $$\begin{array}{c} J\left(\;u^{*}\left(\;t\;\right),v^{*}\left(\;t\;\right)\;\right)=\underset{\Gamma }{\min}\;J\left(\;u\left(\;t\;\right),v\left(\;t\;\right)\;\right). \end{array}$$

The existence of optimal controls follows from standard results in optimal control theory [26].  Theorem 3 follows from Appendix C.

**Theorem 3 .**

Problem (7)–(10) with given initial conditions *S*(0), *I*(0), *I*_*a*_(0), *M*(0), and *R*(0) and fixed final time *T* admits a unique optimal solution (*S* ^*∗*^(*t*), *I* ^*∗*^(*t*), *I*_*a*_^*∗*^(*t*), *M* ^*∗*^(*t*), *R* ^*∗*^(*t*)) associated with an optimal control pair (*u* ^*∗*^, *v* ^*∗*^) on [0, *T*].

The optimal control pair predicted by (5) represents the optimal intervention strategy, given cost constraints, and can be found by the application of the Pontryagin maximum principle [26].

#### 2.3.1. Optimal Control Numerical Results

State system (7) and the adjoint system of differential equations together with the control characterization above from the optimality system are solved numerically using the “forward-backward sweep method (FBSM).” For a detailed discussion on FBSM, we refer the reader to [23]. We set *S*(0) = 0.97, *I*(0) = 0.02, *I*_*a*_(0) = 0.01, and *M*(0) = *R*(0) = 0. Parameters used are in Table 2.

**Figure 2:**
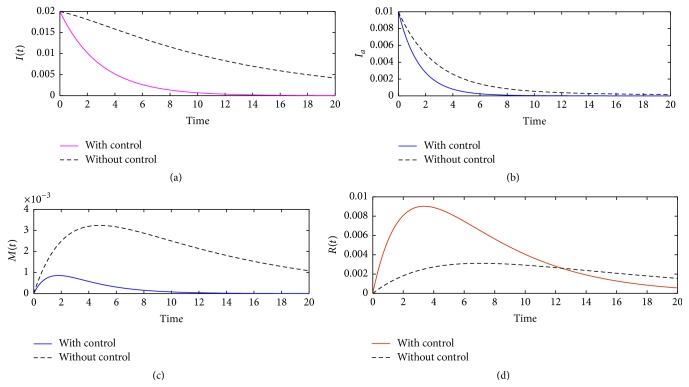
Dynamics of system (C.3) showing the effects of optimal control strategies on eliminating or reducing illicit drug use in the community. The control functions *u*(*t*) and *v*(*t*) were set to 0.95 and *A* = 5 × 10^−7^, *B* = 3 × 10^−7^.

Numerical results in Figure 2 illustrate the role of optimal intervention strategies in controlling illicit drug use in the community. In Figure 2, the balancing coefficients are fixed within the integral expression (8) and the optimal schedule of the two controls over *T* = 20 years is simulated with the following assumed initial population levels: *S* = 0.97, *I* = 0.02, *I*_*a*_ = 0.01, and *M* = *R* = 0. System (7) is used to determine the resultant population dynamics. From the illustrations, it is clear that optimal intervention strategies provide a more effective approach on the elimination or reduction of illicit drug use and mental health cases. In Figure 2(a), we note that in the presence of two optimal intervention strategies it can require 14 years for the population of light illicit drug users to die off. Although interventions which are not time dependent lead to a decrease in cases of light illicit drug use, implemented for 14 years, they may not yield cessation in illicit drug use cases.

In Figure 2(b), we set the initial population level of heavy drug users at 0.01 (1%) of the total population. Although this might be a high figure in reality, it allows us to observe the level of effectiveness of optimal intervention strategies. It is evident in Figure 2 that the presence of the aforementioned optimal intervention strategies can lead to elimination of heavy drug users even if their population is considerably high.

In Figures 2(c) and 2(d), the initial densities of mentally ill and detected population were set to zero. Results here show that in the presence of time dependent interventions mental illness associated with illicit drug use will remain low and will eventual die off after 14 years of implementing the aforementioned optimal intervention strategies. Further, it is evident that more illicit drug users will be detected when time dependent interventions are implemented.

**Figure 3:**
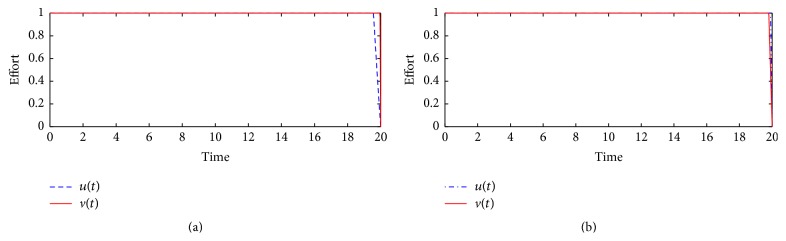
Control profiles for control functions *u*(*t*) = 0.95 and *v*(*t*) = 0.95. In (a) *A* = 5 × 10^−7^ and *B* = 1 × 10^−7^; in (b) *A* = 1 × 10^−7^ and *B* = 5 × 10^−7^.

Simulations in Figure 3 illustrate the feasibility of the control functions *u*(*t*) and *v*(*t*). If the control profile starts at the upper bound and drops on the final time to the origin, then it is regarded as highly feasible, implying that more effort and resources should be devoted to such an intervention strategy for effective problem-solving or control.  Figure 3(a) assumes that parameter *A* associated with control function *u*(*t*) is greater than parameter *B* associated with control function *v*(*t*). This implies that the cost associated with the implementation of control *u*(*t*) is greater than the one associated with control *v*(*t*). For this case, we observe that Figure 3(a) suggests that a slightly higher effort should be devoted to control *v*(*t*), since it is more feasible compared to control *u*(*t*). In Figure 3(b), it is evident that if the cost associated with control *v*(*t*) is higher than the cost associated with control *u*(*t*), then all the optimal intervention strategies are highly feasible.

#### 2.3.2. Efficacy of Optimal Intervention Strategy

The efficacy of an intervention strategy on controlling the generation of new illicit drug users reflects the strength of the strategy to effectively control the drug use problem in the community. In this section, we explore the effectiveness of the aforementioned optimal intervention methods on reducing cumulative population illicit drug users. We define the efficacy function *E*(*t*)

(11) $$\begin{array}{c} E\left(\;t\;\right)=1-\left[\;\frac{I^{*}\left(t\right)+I_{a}^{*}\left(t\right)}{I\left(0\right)+I_{a}\left(0\right)}\;\right], \end{array}$$

where *I* ^*∗*^(*t*) and *I*_*a*_^*∗*^(*t*) denote the optimal solutions associated with the optimal control of the corresponding variable and *I*(0) and *I*_*c*_(0) denote the corresponding initial condition. Function (11) measures the proportional decrease in the number of active illicit drug users imposed by the intervention with controls (*u*, *v*), by comparing the number of active illicit drug users at time *t* with the initial conditions for which there are no controls implemented (*u* = *v* = 0). By construction, *E*(*t*)∈[0,1] for all time *t*. Thus, the upper bound of *E*(*t*) is one.

**Figure 4:**
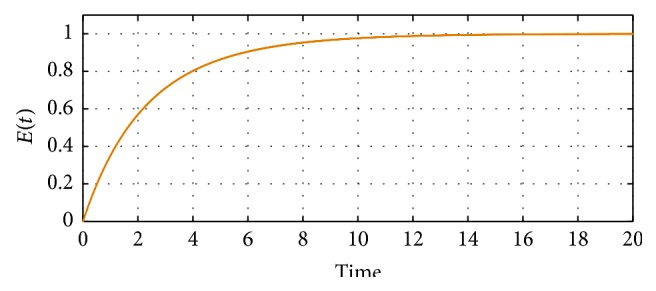
Time series plot demonstrating the efficacy of optimal intervention strategies over a period of 500 days.

Figure 4 demonstrates that optimal interventions may be effective to eliminate illicit drug use in the community after 14 years of implementation. It is worth noting that, after 4 years of implementing these methods, the efficacy level would reach 80% mark; this demonstrates that even for a short time horizon optimal intervention strategies can have a significant impact on controlling illicit drug use in the community.

## 3. Concluding Remarks

About 5% (230 million) of the people of the world's adult population are estimated to have used an illicit drug at least once in 2010 [21]. In this paper, a simple mathematical model to assess the impact of illicit drug use and its adverse health effects is proposed and analyzed. The model is further extended to incorporate two optimal intervention strategies. The goal of the first optimal intervention is tied on reducing the “social influence” between the susceptible and illicit drug users, while the second aims to increase the detection and rehabilitation of illicit drug users. From the illustrations in this study it is clear that time dependent intervention strategies can lead to elimination of illicit drug use in the community. Our analysis also suggests that the implementation of these controls should be devoted to both controls since they are highly feasible for a longer period considered in this study. Although the model is simple and has a couple of simplifying assumptions, qualitative conclusions can be reached in rather broad terms from the simulations and analysis, the kind of conclusions that can generate or provide useful insights into value and effectiveness of time dependent control efforts, aimed at elimination or effective control of illicit drug use and its adverse health problems.

Our model is not exhaustive; a new model that incorporates illicit drug use and age or gender can be developed and used to forecast future trends on illicit drug use.

## Figures and Tables

**Table 1:** Recorded prevalence of illicit drug use in Cape Town, South Africa.

Year	96b	97a	97b	98a	98b	99a	99b	00a	00b	01a	01b	02a
%	20	19	22.1	25	35.1	44.1	50.1	54.2	48.1	57.1	54.3	54.3

Year	02b	03a	03b	04a	04b	05a	05b	06a	06b	07a	07b	08a
%	52.8	58.7	62.9	61.1	66.3	65.6	74.9	69.9	73.2	70.5	70.5	69.4

**Table 2:** Model parameters and their baseline values obtained after fitting data in Appendix A.

Parameter definition	Symbol	Range	Baseline value	Units
Recovery rate	*ω*	0.1–0.9	0.3	Per year
Recruitment rate	*μ*	0.02–0.03	0.02	Per year
Modification factor	*κ*	1–1.85	1.25	—
Transmission rate	*β*	0.31–0.36	0.35	Per year
Detection rate for light drug users	*γ*	0.01–0.14	0.1	Per year
Detection rate for heavy drug users	*ρ*	0.13–0.78	0.35	Per year
Detection rate for mentally ill population	*ϵ*	0.54–0.85	0.6	Per year
Escalation of a light user to a heavy user	*α*	0.01–0.75	0.01	Per year
Drug use-related death for light drug users	*ψ*	0.01–0.05	0.035	Per year
Drug use-related death for heavy drug users	*δ*	0.1–0.18	0.14	Per year
Drug use-related death for mentally ill population	*d*	0.077–0.23	0.2	Per year
Proportion of light users who develop mental illness	*σ*	0.10–0.33	0.05	Per year
Proportion of heavy users who develop mental illness	*ϕ*	0.08–0.09	0.09	Per year

**Table 3:** Sensitivity indices of *ℛ*_*d*_ to parameters for model (2), evaluated at the baseline parameter values given in Table 2.

Parameter	Sensitivity index
*β*	+1
*α*	−0.067
*κ*	0.032
*μ*	−0.38
*ρ*	−0.013
*d*	−0.008
*f*	−0.0075
*ψ*	−0.19
*σ*	−0.059
*γ*	−0.19
*ϕ*	−0.0082

**Table 4:** Recorded prevalence of illicit drug use for Durban, South Africa.

Year	96b	97a	97b	98a	98b	99a	99b	00a	00b	01a	01b	02a
%	27	31	34	35	33	38	35	40	40	41	42	43

Year	02b	03a	03b	04a	04b	05a	05b	06a	06b	07a	07b	08a
%	42	43.7	44.6	42.1	46	48.9	45.4	48.6	46	50.2	55.2	50.5

**Table 5:** Recorded prevalence of illicit drug use for Gauteng, South Africa.

Year	96b	97a	97b	98a	98b	99a	99b	00a	00b	01a	01b	02a
%	—	—	—	31	32	33	37	40	40	46	48	46

Year	02b	03a	03b	04a	04b	05a	05b	06a	06b	07a	07b	08a
%	46	47.8	50.7	49.6	49	53.4	48.2	52.5	52.8	54.1	53	53

**Table 6:** Recorded prevalence of illicit drug use for Mpumalanga, South Africa.

Year	96b	97a	97b	98a	98b	99a	99b	00a	00b	01a	01b	02a
%	—	—	—	—	—	24	24	29	23	30	31	29

Year	02b	03a	03b	04a	04b	05a	05b	06a	06b	07a	07b	08a
%	32	30.9	38.9	36.2	39.2	44.4	45.7	45.5	52.7	56.3	56.7	60.4

## Appendices

### A. Recorded Data on Illicit Drug Use

In this section, we present cumulative prevalence of illicit drug use in South Africa, for the following areas: Durban, Gauteng, and Mpumalanga. The data was adopted from Pluddemann et al. (2008) [8]. We obtained cumulative prevalence after summing the prevalences for the following illicit drugs: cannabis, Mandrax, cocaine, heroin, ecstasy, cocaine, over-the-counter and prescription medicines (OTC/PRE), methamphetamine, and other substances/polysubstance abuse. Key: 96b represents data recorded in 1996 from July to December and 97a represents January to June 1997.

### B. Computation of the Reproductive Number

Following Van den Driessche and Watmough [27] and using the notation defined therein, the matrices *F* and *V* for the new infection terms and the remaining transfer terms are, respectively, given by

(B.1) F=ββκ00,V=μ+α+γ+σ0−αμ+ρ+ϕ+d.

Thus,

(B.2) FV−1=βακ+μ+ρ+ϕ+dα+γ+μ+σ+ψμ+ρ+ϕ+dβμ+ρ+ϕ+d00.

Therefore, spectral radius is

(B.3) $$\begin{array}{c} R_{d}=\frac{\beta \left(\alpha \kappa +\mu +\rho +\phi +d\right)}{\left(\alpha +\gamma +\mu +\sigma +\psi \right)\left(\mu +\rho +\phi +d\right)}. \end{array}$$

### C. Equilibrium States and Their Stability

#### C.1. Global Stability of the Drug-Free Equilibrium

To explore the global stability of *ℰ* ^0^, we consider the Lyapunov function:

(C.1) $$\begin{array}{c} L\left(\;t\;\right)=\left(\;\alpha \kappa +k_{2}\;\right)I\left(\;t\;\right)+\kappa k_{1}I_{a}\left(\;t\;\right). \end{array}$$

Its Lyapunov derivative along the solutions to system (2) is

(C.2) L˙ακ+k2I˙+κk1Ia˙=k1k2βακ+k2k1k2S−1I+κIa≤k1k2βακ+k2k1k2−1I+κIa=I+κIaRd−1

with *k*_1_ = (*μ* + *α* + *γ* + *σ*) and *k*_2_ = (*μ* + *ρ* + *ϕ* + *d*). Thus, $\dot{L}\leq 0$ if *ℛ*_0_ ≤ 1 with $\dot{L}=0$ if and only if *I* = *I*_*a*_ = 0. Therefore, by LaSalle's Invariance Principle [28], every solution to system (2), with initial conditions in *Ω*, approaches *ℰ* ^0^ as *t* → *∞*. That is, (*I*, *I*_*a*_)→(0,0) as *t* → *∞*. Substituting *I* = *I*_*a*_ = 0 in (2) gives *ℰ* ^0^ as *t* → *∞*. Thus, *ℰ* ^0^ is globally asymptotically stable in *Ω* whenever *ℛ*_*d*_ ≤ 1.

#### C.2. Global Stability of the Drug Persistence Equilibrium

Solving system (2) gives

(C.3) $$\begin{array}{c} E^{*}\left\{\begin{array}{cc} S^{*}=\frac{1}{R_{d}}, & \\ I^{*}=\frac{\mu }{k_{1}R_{d}}\left(R_{d}-1\right), & \\ I_{a}^{*}=\frac{\alpha \mu }{k_{1}k_{2}R_{d}}\left(R_{d}-1\right), & \\ M^{*}=\frac{\mu \left(\sigma k_{2}+\alpha \phi \right)}{k_{1}k_{2}k_{3}}\left(R_{d}-1\right), & \\ R^{*}=\frac{\mu \left(\rho \alpha +\gamma k_{3}k_{4}+\epsilon \left(\sigma k_{2}+\alpha \phi \right)\right)}{k_{1}k_{2}k_{3}k_{4}}\left(R_{d}-1\right), & \\ \text{with}\;\;k_{3}=\epsilon +\mu +\delta ,\;\;k_{4}=\mu +\omega . & \end{array}\right. \end{array}$$

Theorem C.1 C.1. If *ℛ*_*d*_ > 1, system (2) is uniformly persistent; namely, there exists a constant *ξ* > 0 such that

(C.4) lim inft→+∞⁡ St>ξ,lim inft→+∞⁡ It>ξ,lim inft→+∞⁡ Iat>ξ;

here, *ξ* is independent with initial data in *Ω*.

Proof When +*∞*, from system (2), we have the following limiting system:

(C.5) S˙=μ−βI+kIaS−μS,I˙=βI+kIaS−k1I,Ia˙=αI−k2Ia.

Observe that we have omitted the last two equations in (2) since the first three equations of system (2) are independent of the variables *M* and *R*. Further, for ease of notation, we will continue to denote the drug-free equilibrium point of system (C.5) as *ℰ* ^0^. Define

(C.6) X=S,I,Ia:  S≥0,  I≥0,  Ia≥0,X0=S,I,Ia:  S≥0,  I≥0,  Ia≥0,∂X0=XX0.

It then suffices to show that (C.5) is uniformly persistent with respect to (*X*, ∂*X*_0_). Firstly, from (C.5), it can be verified that both *X* and *X*_0_ are positively invariant. Clearly, ∂*X*_0_ is relatively closed in *X* and system (C.5) is point dissipative. Consider the following set using solutions (*S*(*t*), *I*(*t*), *I*_*a*_(*t*)) of system (C.5):

(C.7) G∂=S0,I0,Ia0:  St,It,Iat∈∂X0,  ∀t≥0.

Now we prove that

(C.8) $$\begin{array}{c} G_{\partial }=\left\{\;\left(\;S,0,0\;\right)\text{: }S\geq 0\;\right\}. \end{array}$$

Assume that (*S*(0), *I*(0), *I*_*a*_(0)) ∈ *G*_∂_. It suffices to show that *I*(*t*) = *I*_*a*_(*t*) = 0 for all *t* ≥ 0. If not, then there exists *t*_0_ ≥ 0 such that (*I*(*t*_0_) > 0, *I*_*a*_(*t*_0_) = 0) or (*I*(*t*_0_) = 0, *I*_*a*_(*t*_0_) > 0). For (*I*(*t*_0_) > 0, *I*_*a*_(*t*_0_) = 0), we have

(C.9) $$\begin{array}{c} \dot{I_{a}}=\alpha I\left(\;t_{0}\;\right)>0. \end{array}$$

It follows that there is *ϵ*_0_ > 0 such that *I*_*a*_(*t*) > 0 for *t*_0_ < *t* < *t*_0_ + *ϵ*_0_. Clearly, we can restrict *ϵ*_0_ > 0 small enough such that *I*(*t*) > 0 for *t*_0_ < *t* < *t*_0_ + *ϵ*_0_. This means that (*S*(*t*), *I*(*t*), *I*_*a*_(*t*))∉∂*X*_0_ for *t*_0_ < *t* < *t*_0_ + *ϵ*_0_, which contradicts the assumption that (*S*(0), *I*(0), *I*_*a*_(0)) ∈ *G*_∂_. For other cases, it can also be easily verified that they contradict the assumption that (*S*(0), *I*(0), *I*_*a*_(0)) ∈ *G*_∂_. Thus, (C.8) holds. Note that *ℰ* ^0^ is globally asymptotically stable in Int *G*_∂_. Moreover, *ℰ* ^0^ is an isolated invariant set in *X*, every orbit in *G*_∂_ converges to *ℰ* ^0^, and *ℰ* ^0^ is acyclic in *G*_∂_. By Theorem 4.6 in [29], we only need to show that *W* ^*S*^(*ℰ* ^0^)∩*X*_0_ = *∅* if *ℛ*_*d*_ > 1. Now, we prove that *W* ^*S*^(*ℰ* ^0^)∩*X*_0_ ≠ *∅*. Then, there exists a positive solution $\left(\tilde{S}(t),\tilde{I}(t),{\tilde{I}}_{a}(t)\right)$ with $(\tilde{S}(0),\tilde{I}(0),{\tilde{I}}_{a}(0))\subset X_{0}$, such that $(\tilde{S}(t),\tilde{I}(t),{\tilde{I}}_{a}(t))\to {ℰ}^{0}$ as *t* → +*∞*. Since *ℛ*_*d*_ > 1, we choose *η* > 0 small enough such that

(C.10) $$\begin{array}{c} \left(\;1-\eta \;\right)R_{d}>1. \end{array}$$

Thus, when *t* is sufficiently large, we have $S^{0}-\eta \leq \tilde{S}(t)\leq S^{0}+\eta$, $0\leq \tilde{I}(t)\leq \eta$, $0\leq {\tilde{I}}_{a}(t)\leq \eta$, and

(C.11) I˙=βI+kIaS0−η−k1I,Ia˙=αI−k2Ia.

By the comparison principle, it follows that $\tilde{I}(t)\to +\infty$, ${\tilde{I}}_{a}(t)\to +\infty$ as *t* → +*∞*, which contradicts $\tilde{I}(t)\to 0$, ${\tilde{I}}_{a}(t)\to 0$ as *t* → +*∞*. This proves *W* ^*S*^∩*X*_0_ = *∅*. Since *W* ^*S*^(*ℰ* ^0^)∩*X*_0_ = *∅*, ⋃_*x*∈*G*_∂__ *ω*(*x*) = {*ℰ* ^0^}, *ℰ* ^0^ is an isolated invariant set in *X*, and *ℰ* ^0^ is acyclic in *G*_∂_, and by Theorem 4.6 in [29], hence we are able to conclude that system (C.5) is uniformly persistent with respect to (*X*_0_, ∂*X*_0_). Then, system (2) is uniformly persistent.

We proceed to investigate the global stability of *ℰ* ^*∗*^.

Theorem C.2 C.2 (see [30]). Let *x* ↦ *f*(*x*) ∈ *ℝ* ^*n*^ be a *C* ^1^ function for *x* in an open set *D* ⊂ *ℝ* ^*n*^. Consider the differential equation

(C.12) $$\begin{array}{c} \dot{x}=f\left(\;x\;\right). \end{array}$$

Denote by *x*(*t*, *x*_0_) the solution to (C.12) such that *x*(0, *x*_0_) = *x*_0_. A set *K* is said to be absorbing in *D* for (C.12) if *x*(*t*, *K*_1_)⊂ for each *K*_1_ ⊂ *D* and *t* is sufficiently large. If the following conditions are satisfied, then the unique equilibrium $\bar{x}$ is globally asymptotically stable:

(1) There exists a compact absorbing set *K* ⊂ *D* and (C.12) has a unique equilibrium in $\bar{x}$ in *D*.
(2) $\bar{x}$ is locally asymptotically stable.
(3) System (C.12) satisfies a Poincare-Bendixson Property.
(4) Each periodic orbit of (C.12) in *D* is asymptotically orbitally stable.

Theorem C.3 C.3. The trajectory of any nonconstant periodic solution of system (2), if it exists, is asymptotically orbitally stable with asymptotic phase.

On investigating the global stability of *ℰ* ^*∗*^, the equations for the mentally ill individuals and the recovered population can be omitted. Thus, system (2) reduces to

(C.13) S˙=μ−βI+kIaS−μS,I˙=βI+kIaS−k1I,Ia˙=αI−k2Ia.

We study system (C.13) in the closed set:

(C.14) $$\begin{array}{c} \Phi =\left\{\;\left(\;S,I,I_{a}\;\right)\in R_{+}^{3}:0\leq S+I+I_{a}\leq N\;\right\}, \end{array}$$

where Φ is positively invariant with respect to (C.13). The corresponding associated second compound matrix [31, 32] is given by

(C.15) $$\begin{array}{c} A^{\left[2\right]}=\left(\begin{array}{ccc} -\mu -k_{1}-\beta \left(I+\kappa I_{a}\right)+\beta S & \beta \kappa S & \beta \kappa S\\ \alpha & -\mu -k_{2}-\beta \left(I+\kappa I_{a}\right) & -\beta S\\ 0 & \beta \left(I+\kappa I_{a}\right) & -k_{1}-k_{2}+\beta S \end{array}\right). \end{array}$$

Then, the second compound system defined along the periodic solution (*S*(*t*), *I*(*t*), *I*_*a*_(*t*)) of system (C.13) is given by

(C.16) Xt˙=−μ+k1+βI+κIa−SXt+βκSYt+Zt,Yt˙=αXt−μ+k2+βI+κIaYt−βSZt,Zt˙=βI+κIaYt+βS−k1+k2Zt.

Based on Theorem C.2, if we can prove that system (C.13) is asymptotically stable, then the periodic solution is asymptotically orbitally stable with asymptotic phase. We define the Lyapunov function

(C.17) $$\begin{array}{c} V\left(\;X,Y,Z,S,I,I_{a}\;\right)=\sup\left\{\;\left|\;X\;\right|,\frac{I}{I_{a}}\left(\;\left|\;Y\;\right|+\left|\;Z\;\right|\;\right)\;\right\}. \end{array}$$

From condition (2), we have that there exists a constant *c*_0_ > 0 such that

(C.18) $$\begin{array}{c} V\left(\;X,Y,Z,S,I,I_{a}\;\right)\geq c_{0}\sup\left\{\;\left|\;X\;\right|,\left|\;Y\;\right|,\left|\;Z\;\right|\;\right\}, \end{array}$$

for any (*X*, *Y*, *Z*) ∈ *ℝ*_+_^3^ and any periodic solution (*S*, *I*, *I*_*a*_) ∈ Φ. Let us estimate the right derivative of *V* along a solution (*X*(*t*), *Y*(*t*), *Z*(*t*)) of system (C.16):

(C.19) D+Xt≤−μ+k1+βI+κIa−SXt+βκSYt+Zt,D+Yt≤αXt−μ+k2+βI+κIaYt−βSZt,D+Zt≤βI+κIaYt+βS−k1+k2Zt,D+IIaY+Z=I˙I−Ia˙IaIIa+IIaD+Y+Z≤I˙I−Ia˙IaIIa+IIaD+Y+Z+IIaαX−2μ+ρ+ϕ+dY+Z=αIIaX+I′I−Ia′Ia−2μ+ρ+ϕ+dIIaY+Z.

We then need to estimate the following two functions:

(C.20) g1t=−2μ−α+γ+ψ+δ−βI+κIa+βS+βκIaSI,g2t=αIIa−2μ−ρ+ϕ+d+I′I−Ia′Ia.

From system (C.13), we have

(C.21) I˙I=βS+βκIaSI−μ+α+γ+σ+ψ,Ia˙Ia=αIIa−μ+ρ+ϕ+d.

Thus,

(C.22) g1=I˙I−μ+ψ−βI+κIa≤I˙I−μ,g2≤I˙I−μ.

Therefore,

(C.23) $$\begin{array}{c} m\left(\;Q\;\right)\leq \frac{\dot{I}}{I}-\mu . \end{array}$$

Denote the period of the periodic solutions (*S*(*t*), *I*(*t*), *I*_*a*_(*t*)) by *τ*. We have

(C.24) $$\begin{array}{c} \overset{\tau }{\underset{0}{\int }}\left[\;\frac{\dot{I}}{I}-\mu \;\right]dt\leq {\left.\;\ln I\left(\;t\;\right)\;\right|}_{0}^{\tau }-\mu \tau =\ln I\left(\;t\;\right)-\mu \tau <0. \end{array}$$

Thus, system (C.13) is asymptotically stable. Then, the periodic solutions (*S*(*t*), *I*(*t*), *I*_*a*_(*t*)) are asymptotically orbitally stable with asymptotic phase.

Proof of Theorem 3. System (2) is converted into an equivalent problem, namely, the problem of minimizing the Hamiltonian *H* given by

(C.25) H=I+Ia+Au2+Bv2+λ1μ−1−uλS−μS+λ21−uλS−α+γv+σ+μ+ψI+λ3αI−ϕ+ρv+μ+dIa+λ4σI+ϕIa−μ+ϵv+δM+λ5vγI+ρIa+ϵM−μ+ωR,

where *λ*_*i*_, for *i* = 1,2,…, 5, are the adjoint functions associated with their respective states. Note that, in (C.25), each adjoint function multiplies the right-hand side of the differential equation of its corresponding state function. The first terms in *H* come from the integrand of the objective functional. Given an optimal control pair (*u* ^*∗*^, *v* ^*∗*^) and corresponding states (*S* ^*∗*^, *I* ^*∗*^, *I*_*a*_^*∗*^, *M* ^*∗*^, and *R* ^*∗*^), there exist adjoint functions satisfying

(C.26) λ1t˙=μλ1+1−uβI+κIaλ1−λ2,λ2t˙=−1+ψ+μλ2+σλ2−λ3+αλ2−λ4+vγλ2−λ5+1−uβSλ1−λ2,λ3t˙=−1+μ+dλ3+ϕλ3−λ4+vρλ3−λ5+1−uβκλ1−λ2S,λ4t˙=μλ4+vϵλ4−λ5,λ5t˙=μ+ωλ5,

with transversality conditions, *λ*_*i*_(*T*) = 0, for *i* = 1,2,…, 5. Observe that the right-hand side of *λ*_1_ differential equation is −∂*H*/∂*S*, and similarly for the other adjoint functions. The final time boundary conditions (transversality conditions) are zero since there is no dependence on the states at the final time in the objective functional. Furthermore, the optimal controls are characterized by

(C.27) u∗t=min⁡max⁡0,βSI+κIaλ2−λ12A,1,v∗t=min⁡max⁡0,γλ2−λ5I+ρλ3−λ5Ia+ϵλ4−λ5M2B,1.

The control characterization for *u* ^*∗*^ is obtained on ∂*H*/∂*u* = 0 whenever 0 < *u* ^*∗*^(*t*) < 1 and taking bounds into account, similar to the other controls.
